# The impact of pre‐analytical variations on biochemical analytes stability: A systematic review

**DOI:** 10.1002/jcla.23551

**Published:** 2020-09-01

**Authors:** Mehdi Hedayati, S. Adeleh Razavi, Seti Boroomand, Sima Kheradmand Kia

**Affiliations:** ^1^ Cellular and Molecular Endocrine Research Center Research Institute for Endocrine Sciences Shahid Beheshti University of Medical Sciences Tehran Iran; ^2^ Department of Research and Development (R&D) Saeed Pathobiology & Genetics Laboratory Tehran Iran; ^3^ Vancouver General Hospital Vancouver British Columbia Canada; ^4^ Laboratory for Red Blood Cell Diagnostics Sanquin, Amsterdam The Netherlands

**Keywords:** biochemical analytes, plasma, pre‐analytical variations, serum, stability, whole blood

## Abstract

**Objective:**

A common problem in clinical laboratories is maintaining the stability of analytes during pre‐analytical processes. The aim of this study was to systematically summarize the results of a set of studies about the biochemical analytes stability.

**Methods:**

A literature search was performed on the Advanced search field of PubMed using the keywords: “(stability) AND (analytes OR laboratory analytes OR laboratory tests OR biochemical analytes OR biochemical tests OR biochemical laboratory tests).” A total of 56 entries were obtained. After applying the selection criteria, 20 articles were included in the study.

**Results:**

In the 20 included references, up to 123 different analytes were assessed. The 34 analytes in order of the most frequently studied analytes were evaluated: Alanine aminotransferase, aspartate aminotransferase, potassium, triglyceride, alkaline phosphatase, creatinine, total cholesterol, albumin, lactate dehydrogenase, sodium, calcium, γ‐glutamyltransferase, total bilirubin, urea, creatine kinase, inorganic phosphate, total protein, uric acid, amylase, chloride, high‐density lipoprotein, magnesium, glucose, C‐reactive protein, bicarbonate, ferritin, iron, lipase, transferrin, cobalamin, cortisol, folate, free thyroxine, and thyroid‐stimulating hormone. Stable test results could be varied between 2 hours and 1 week according to the type of samples and/or type of blood collection tubes on a basic classification set as refrigerated or room temperature.

**Conclusions:**

Biochemical analytes stability could be improved if the best pre‐analytical approaches are used.

## Graphical abstract

To mimic the pre‐analytical sample handling takes longer than usual, we performed a systematic review study and evaluated the effect of different storage time at different storage temperatures on the stability of 34 common biochemical analytes.

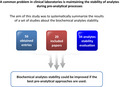

**Abbreviations**
ACTH: adrenocorticotrophic hormone
AL: albumin
ALP: alkaline phosphatase
ALT: alanine aminotransferase
AM: amylase
ApoA1: apolipoprotein A1
ApoB: apolipoprotein B
APTT: Activated partial thromboplastin time
AST: aspartate aminotransferase
AT: anti‐thrombin III
B12: cobalamin
BIC: bicarbonate
BSAP: bone specific alkaline phosphatase
BUN: urea
C3: complement component‐3
C4: complement component‐4
Ca^2+^: calcium
CEA: carcinoembryonic antigen
CI: chloride
CK: creatine kinase
COR: cortisol
CP: C‐peptide
CREA: creatinine
CRP: C‐reactive protein
CTELO: C‐telopeptide
DB: direct bilirubin
DHEAS: dehydroepiandrosterone sulfate
E:C: eosinophil
E2: estradiol
FERR: ferritin
FG: fibrinogen
FOL: folate
FRU: fructosamine
FSH: follicle‐stimulating hormone
FT3: free triiodothyronine
FT4: free thyroxine
GGT: γ‐glutamyltransferase
Gh: growth hormone
GLU: glucose
HAP: haptoglobin
HDL: high‐density lipoprotein
HG: hemoglobin concentration
hs‐TnT: high sensitivity troponin T
HT: hematocrit
IGF‐1: insulin‐like growth factor
INS: insulin
Ir: iron
K^+^: potassium
L:C: lymphocyte counts
LAC: lactate
LDH: lactate dehydrogenase
LDL: low‐density lipoprotein
LH: luteinizing hormone
LIP: lipase
M:C: monocyte
MCH: mean corpuscular hemoglobin
MCHC: mean corpuscular hemoglobin concentration
MCV: mean corpuscular volume
MG: myoglobin
Mg^2+^: magnesium
N:C: neutrophil
Na^+^: sodium
NT‐pro BNP: N‐terminal pro brain natriuretic peptide
OST: osteocalcin
P:C: platelet counts
Pi: inorganic phosphate
PLIP: phospholipids
PROG: progesterone
PROL: prolactin
PSA: prostate‐specific antigen
PT: prothrombin time
PTH: parathyroid hormone
RBC: red blood cells counts
Se: selenium
SHBG: sex hormone binding globulin
STFR: soluble transferrin receptor
TAC: total antioxidant capacity
TB: total bilirubin
TC: total cholesterol
TEST: testosterone
TF: transferrin
TG: triglyceride
TnT: troponin T
TP: total protein
TSH: thyroid‐stimulating hormone
UA: uric acid
vit D: vitamin D
WBC: white blood cell counts
α1‐MG: α1‐macroglobulin
α2‐MG: α2‐macroglobulin
β‐MG: β‐macroglobulin
γ‐MG: γ‐macroglobulin

## 1 INTRODUCTION

Clinical chemistry and laboratory medicine are fundamental parts of the diagnosis of human diseases. It is clear that the minimized interval between sample collection and processing is the best strategy to prevent changes in analytes activities and concentrations. However, some of the recommendations from guidelines^1^, ^2^ are difficult to apply in routine practice. Therefore, a common problem in clinical laboratories is maintaining the stability of serum/plasma analytes during pre‐analytical sample handling (collection and preparations) and next, during post‐analytical sample handling (storage time and temperature).

Due to increased separation process and decreased hemolysis, many clinical laboratories for routine analytes have been using plasma or serum separator tubes. The gel used in these tubes is relatively inert; however, it may affect analyte concentrations or stability. More importantly, commonly ordered analytes (eg, potassium and phosphate) are known to be sensitive to delayed centrifugation and temperature. Thus, type of blood collection tube, the time interval between sample collection and analysis, and finally the time and temperature at which the samples are stored constitutes important variables that may affect analysis results and may lead to wrong clinical decisions.^3^

Stability limits should be defined for each analyte and sample and be used as a cause of rejection before processing the sample. The stability limit for an analyte is defined as the point in time when the percent deviation (PD%) of an analyte reaches the maximum allowable error. Although a countless number of experimental studies have assessed the stability of most laboratory analytes in various conditions, there are few clinical guidelines with general recommendations for the laboratories. On the other hand, among the existing studies, there are different or even inconsistent results due to the lack of standard experimental designs and wide variability in maximum permissible instability (MPI) specifications.^4^

Alongside these challenges, the evolving scenario of consolidation of smaller laboratories into larger ones has become more popular nearly all over the world. One of the leading consequences of the core labs creation is that patients’ samples affect the extra‐preanalytical factors such as different transportation conditions, prolonged storage at high or low temperature, and improper handling. On the other hand, research biobanks usually aimed to perform observational epidemiological studies, and/or interventional projects, collect biological samples from a large population and freeze the samples for long‐term storage for future analyses. Therefore, to mimic the pre‐analytical sample handling takes longer than usual in core laboratories or in research biobanks, we performed a systematic review study and evaluated the effect of storage time from 45 min to 12 months at different storage temperatures on the stability of biochemical analytes. To our knowledge, this study is the first of its type which systematically evaluated the effect of storage conditions on more than 30 biochemical analytes testing.

## 2 MATERIALS AND METHODS

### 2.1 Search strategy

A PubMed Advanced Search was performed without any time limit using the following keywords in the Title Field: “(stability) AND (analytes OR laboratory analytes OR laboratory tests OR biochemical analytes OR biochemical tests OR biochemical laboratory tests).” A total of 56 entries were obtained. The acceptable articles were selected by the two authors by checking the titles and abstracts.

### 2.2 Study selection

**FIGURE 1:**
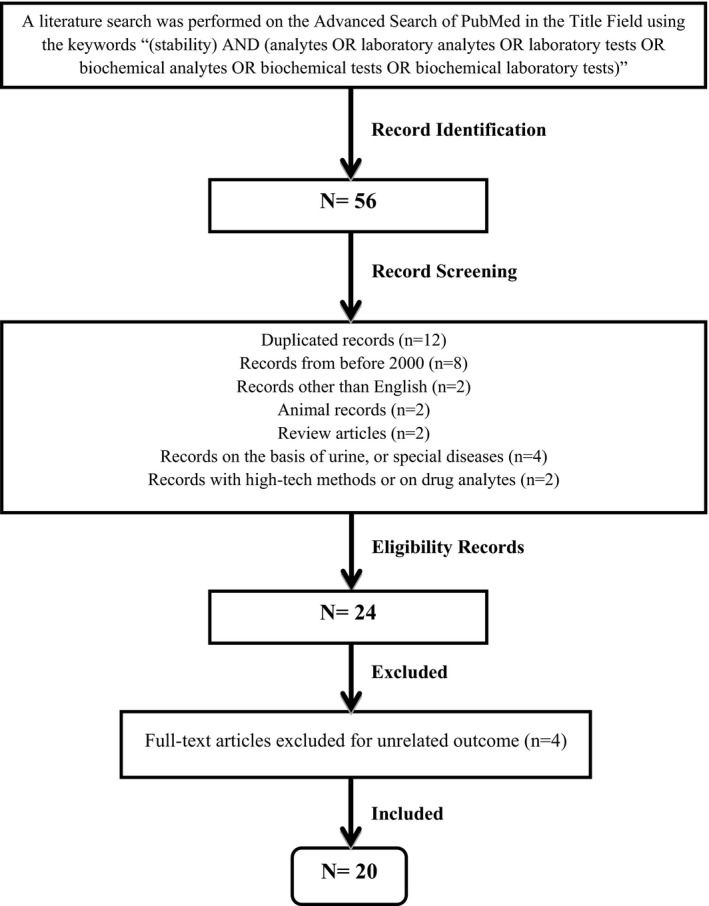
Review flowchart for selection of included papers

The process of study selection was as follows: (1) exact duplicate articles were removed by EndNote tools; (2) articles other than English were removed; and (3) irrelevant studies according to the exclusion criteria (Figure 1) made by the authors were excluded. After applying the selection criteria, 20 articles^5^, ^6^, ^7^, ^8^, ^9^, ^10^, ^11^, ^12^, ^13^, ^14^, ^15^, ^16^, ^17^, ^18^, ^19^, ^20^, ^21^, ^22^, ^23^, ^24^ were included in the study.

### 2.3 Stability conditions

In order to classify reviews of the papers, a variables checklist including the following items was defined: (1) sample size of the study; (2) sample type of the study; (3) sample container; (4) instruments used in the study; (5) metabolites; (6) baseline value used in the study; (7) tested values investigated in the study; and (8) statistical analysis. These items were precisely reviewed and recorded for 20 included articles (Table 1).

**TABLE 1:** Characteristics of eligible studies

Reference		Sample size	Sample type	Sample container	Instruments	Metabolites*	Baseline value	Tested values	Statistical analysis
1	Boyanton, et al ^5^	10 nonfasting volunteers	Whole blood, serum, plasma	Vacutainer® Serum Separator Tubes with clot activator (BD) & Vacutainer Plasma Separator Tubes with lithium heparin (BD)	Dade Dimension RxL & Gem Premier 2.2 Blood Gas Analyzer & Hitachi 747	AL, ALP, ALT, AST, BIC, BUN, Ca^2+^, CK, Cl, CREA, DB, GGT, GLU, HG, K^+^, LAC, LDH, Mg^2+^, Na^+^, PCO2, pH, Pi, PO2, TB, TC, TG, TP, UA	Centrifuged samples within 30 min at 25°C	I) Uncentrifuged samples until 4, 8, 16, 24, 32, 40, 48, 56 h after collection stored at 25°C; II) Double centrifuged samples stored for 4, 8, 16, 24, 32, 40, 48, 56 h at 25°C	ANOVA and SCL
2	Clark, et al ^6^	12 nonfasting volunteers	Plasma	Vacutainers of blood containing potassium EDTA with aprotinin (BD)	Synchron LX20 autoanalyzer	AL, ALT, ApoA1, ApoB, AST, CK, CREA, GGT, HDL, LDL, TC, TG, TP	Immediately centrifuged samples at 21°C	Uncentrifuged samples until 1, 2, 3, 4, or 7 days after collection at two storage temperature, 21°C and 4°C	PD% and Log‐linear regression
3	Cuhadar, et al ^7^	15 fasting volunteers	Serum	Vacuette® Tube Serum No Gel & Vacuette® Tube Serum Gel Separator	Abbott Aeroset	AL, ALP, ALT, AST, BUN, CK, CREA, GGT, GLU, HDL, LDH, TB, TC, TG, TP, UA	Centrifuged samples within 30 min at 24°C	Serum samples in contact with gel and without gel stored at 24°C or 4°C for time periods of 6, 12, 18, 24, 30, 36, 48, 72 h, and 1 week	Non‐parametric Friedman test for repeated measures, Wilcoxon signed‐rank test with a Bonferroni correction, and PD%
4	Dupuy, et al ^8^	6 healthy volunteers & 9 hospitalized patients	Plasma	Plastic BD Vacutainer® lithium heparin tube & plastic BD Barricor™ plasma tube (BD)	Cobas 8000© system including c701 and c502 module (for biochemical analytes) and e602 module (for immunoassays)	ALP, ALT, AST, CRP, hs‐TnT, K^+^, LDH, Na^+^, NT‐pro BNP	Centrifuged samples within 30 min at 20°C	Plasma samples stored in two different types of tubes at 4°C for time periods of 4, 24, and 72 h.	Passing‐Bablok regression, Mann Whitney U test, Bland‐Altman plot, PD%, TCL
5	Dupuy, et al ^9^	6 healthy volunteers & 4 hospitalized patients	Serum, plasma	Plastic Vacuette© lithium heparin tubes & plastic serum tubes (BD)	Cobas 8000© system including c701 and c502 module (for biochemical analytes) and e602 module (for immunoassays)	Lithium heparin tubes: ALP, ALT, AST, BIC, BUN, Ca^2+^, CK, Cl, CREA, CRP, FERR, GGT, hs‐TnT, K^+^, LDH, LIP, MG, Mg^2+^, Na^+^, NT‐pro BNP, Pi, TB, UA Serum tubes: ApoA1, ApoB, C P, HDL, INS, OST, PTH, TC, TG	Serum: centrifuged samples after 30 min at 20‐25°C. Plasma: centrifuged samples at 20°C	For whole blood analyte stability, uncentrifuged samples until 2, 4, 6, 8, 12, 24 h after collection at RT storage temperature. For plasma or serum analyte stability, centrifuged samples stored at 4°C for 2, 4, 6, 8, 12, 24, 48 h.	PD%, TCL
6	Gawria, et al ^10^	Part I: 20 hospitalized patients; part II: 102 outpatients	Plasma	Part I: lithium heparin tubes with gel (Vacutainer® PST™, BD) & lithium heparin tubes with a mechanical separator consisting of a flexible elastomer part connected to a firm plastic part (Vacutainer® Barricor™, BD). Part II: lithium heparin tubes from three types including Vacutainer® PST™ gel separator (BD), Vacutainer® Barricor™(BD), and Vacuette® gel separator (Greiner Bio‐One).	Cobas c701	AST, GLU, homocysteine, K^+^, LDH, Mg^2+^, Pi	Centrifuged samples within 15‐30 min in reference tube at 20°C	Plasma samples stored in different types of tubes at 4°C and analyzed after 6, 24, 48, and 72 hours.	PD%, Part I: 2‐sided paired Student´s t test with Bonferroni's correction, Friedman's ANOVA. Part II: Spearman rank order correlation, linear regression model
7	Haslacher, et al ^11^	21 donors as case group, 22 donors as control group	Serum	GBO® Vacuette Serum tubes with Z‐Clot activator (Greiner Bio‐One)	Cobas® 8000 modular analyzer series	AL, ALP, ALT, AM, AST, BUN, Ca^2+^, Cholinesterase, CK, CREA, CRP, FERR, GGT, HDL, Ir, K^+^, LDH, LIP, Na^+^, Pi, TB, TC, TF, TG, TP, UA	Centrifuged samples after > 30 min at RT which were stored in ≤ −75 ºC for 7, 14, 21 days	Centrifuged samples undergone 10, 20, 30 temperature fluctuation events within 7, 14, 21 days	Pearson's chi‐square‐tests, general linear repeated measures models (GLM‐RM), PD%, Non‐linear regression
8	Jackson, et al ^12^	40 healthy volunteers	Serum, plasma and urine	The authors referred to their previous study.	Not mentioned	AL, ALP, ALT, AM, AST, Basophils, BIC, BUN, Ca^2+^, CK, CK MB fraction, Cl, CREA, CRP, E:C, FG, GGT, GLU, GLU (F. Oxalate), HbA1c, HDL, HG, HT, INS, K^+^, L:C, M:C, MCH, MCHC, MCV, Mg^2+^, N:C, Na^+^, Pi, P:C, RBC, TB, TC, TG, TP, UA, Urinary (Ca^2+^, K^+^, Na^+^, urea), WBC	Time 0	I) Samples stored for 12, 24, 36 h; II) Samples undergone freeze‐thaw cycling between 0 and 24 h.	Random effects linear regression, PD%
9	Kachhawa, et al ^13^	10 fasting outpatients	Serum	Plastic Vacuette® serum tubes (BD Vacutainer® serum; BD)	Olympus AU 400 auto analyzer & Roche AVL electrolyte analyzer	AL, ALP, ALT, AM, AST, BUN, Ca^2+^, CREA, DB, K^+^, Na^+^, Pi, TB, TC, TG, TP, UA	Centrifuged samples within 30 min at RT	Samples stored at −20°C for 7, 15 or 30 days	ANOVA, SCL
10	Kocijancic, et al ^14^	90 subjects including of the patients on oral anticoagulant therapy and healthy controls	Serum	Two serum vacuum tubes with clot activator and gel separator: Tube I: BD Vacutainer® Serum‐Separating Tube II Advance Tube (SST) & Tube II: BD Vacutainer® Rapid Serum Tube (RST) (BD)	Olympus AU2700	ALT, AST, Ca^2+^, GLU, K^+^, LDH	Centrifuged samples at 20°C	Samples collected in two different types of tubes and stored at 4°C for 24 h.	Paired t test, Wilcoxon Matched‐Pairs Rank, Passing‐Bablok and Bland‐Altman plots, B, PD%
11	Leino, et al ^15^	50 fasting volunteers	Whole blood	VenoSafeTM lithium heparin gel tubes (Terumo Europe)	Roche Modular PPEE analyzer	AL, ALP, ALT, AM, B12, BUN, C P, Ca^2+^, CEA, CK, Cl, COR, CREA, CRP, free PSA, FT4, GGT, HAP, INS, Ir, K^+^, LDH, Mg^2+^, Na^+^, Pi, PSA, PTH, rheumatoid factor, STFR, TB, TC, TF, TG, TnT, TSH, UA	Centrifuged samples within 0.5 h at 18°C	Centrifuged samples after 6 h storage at 22°C and 8°C	ANOVA or Kruskal‐Wallis test, SCL
12	Mathew, et al ^16^	6 healthy volunteers	Serum	Plastic BD Vacutainer® SST™ Plus Blood Collection Tubes (BD) & cryogenic polypropylene vials (Corning Life Sciences)	Not mentioned	1,25‐dihydroxyvitamin D, AL, ALP, ALT, AST, BSAP, ceruloplasmin, Cl, COR, CREA, FERR, FOL, HDL, homocysteine, Ir, K^+^, LDL, methylmalonic acid, Na^+^, OST, osteoprotegerin, retinol‐binding protein, STFR, TC, TF, TG, TP, transthyretin, vit D, α1‐MG, α2‐MG, β‐MG, γ‐MG	Not mentioned	Serum samples collected in two different types of tubes (cryogenic vials with no gel contact or SST tubes with gel barrier contact) stored at −80°C for 12 month	ANOVA
13	Monneret, et al ^17^	28 healthy volunteers, 21 hospitalized patients	Whole blood and plasma	Lithium heparin tubes (Greiner Bio‐One)	Modular P800® analyzer	AL, ALP, ALT, AM, AST, BIC, BUN, Ca^2+^, CK, Cl, CREA, GGT, HAP, K^+^, LDH, LIP, Mg^2+^, Na^+^, Pi, TB, TC, TG, TP, UA	Centrifuged samples after exactly 2 h at 21.3 ± 1.8°C for whole blood samples. Centrifuged samples after 2 h ± 18 min for plasma samples.	For whole blood analyte stability, centrifuged samples after 3, 4, 5, and 6 h at RT. For plasma analyte stability, baseline centrifuged samples stored at RT for 2, 4, 6 h.	PD%, ACL, RCV
14	Nielsen, et al ^18^	Total sample size was 156 hospitalized patients, but each analyte was analyzed on 32‐60 samples.	Plasma	Lithium heparin gel tubes (Vacutainer, BD Biosciences)	Dimensions Vista®	AL, ALT, AM, B12, Ca^2+^, CREA, CRP, FERR, FOL, FT3, FT4, GGT, HAP, HDL, Ir, K^+^, LDH, Na^+^, TB, TC, TF, TG, TSH	Centrifuged samples within an hour at RT	Decapped plasmas stored at 20‐25°C for 2, 4, 6, 8, and 10 h	SEM, 90%CI, B, TE
15	Oddoze, et al ^19^	50 apparently healthy volunteers	Whole blood, plasma, and serum	Specific BD Vacutainer™ blood collection tubes including 1‐ for biochemistry analyses: plain glass tubes; serum separator tubes with clot activator BD SST™ II and lithium heparin tubes; 2‐ for glucose and lactate: sodium fluoride tubes; 3‐ for hematology analysis: K3 EDTA tubes; 4‐ for coagulation analysis: CTAD tubes; 5‐ for hormonology analysis: plain glass tubes, BD SST™ II and K3 EDTA tubes.	Modular® PP, BNProspec®, RxLDimension®, G5®, Advia 120®, STAR®, Cobas® 6000 e601, and Liaison®	ACTH, AL, ALP, ALT, AM, AMG, ApoA1, ApoB, APTT, AST, AT, B12, BIC, BUN, C P, Ca^2+^, CK, Cl, COR, CREA, CRP, CTELO, DHEAS, E:C, E2, Factor II, Factor V, Factor X, FG, FOL, FRU, FSH, FT3, FT4, GGT, Gh, GLU, HAP, HbA1c, HDL, HG, HT, IGF‐1, INS, Ir, K^+^, L:C, LAC, LDH, LDL, LH, LIP, M:C, MCH, MCHC, MCV, Mg^2+^, MG, N:C, Na^+^, OST, Pi, P:C, PLIP, PROG, PROL, PT, PTH, RBC, SHBG, STFR, TB, TC, TEST, TF, TG, TP, TSH, UA, vit D, WBC	Centrifuged samples after 30 min at 20°C for serum samples. Immediately centrifuged samples at 20°C for plasma samples.	For whole blood analyte stability, centrifuged samples after 2, 4, 6, 24, 72 h either at 4°C or RT. For plasma or serum analyte stability, centrifuged samples (plasma: immediately; serum: 30 min after phlebotomy) stored either at 4°C or RT for 2, 4, 6, 24, 48, and 72 h.	PD%, TCL
16	Shimizu, et al ^20^	7 healthy volunteers	Serum	Conical centrifuge tube (Corning Inc), then CryoTubes with an outer cap (Nunc: Thermo Fisher Scientific)	Beckman Coulter AU480 analyzer	ALT, ALP, AM, AST, C3, C4, CK, GGT, HDL, LDH, LDL, TB, TG	Centrifuged samples stored at −80°C	Centrifuged samples stored at − 30, −20, −10, 0, 4, and 25°C for 1, 3, 7, 14, 28, and 56 days	Wilcoxon signed‐rank test with Bonferroni correction
17	Tanner, et al ^21^	30 healthy volunteers	Whole blood	Gel separator tubes (BD Vacutainer Systems SSTTMII)	Roche Modular PPE analyzer and Abbott Architect i4000 Analyser & DPC Immulite 2000 analyzer and Diasorin equilibrium radioimmunoassay	AL, ALP, ALT, AST, B12, BUN, Ca^2+^, CK, COR, CREA, CRP, E2, FERR, FSH, FT3, FT4, GGT, GLU, Ir, K^+^, LDH, LIP, Mg^2+^, Na^+^, Pi, PSA, TB, TC, TF, TG, TnT, TP, TSH, UA, vit D	Centrifuged samples within 0.5 h at 18‐25°C	Uncentrifuged samples until 4, 12, 24 h after collection stored at 15, 25, or 35°C	Two‐way repeated measures analysis of variance, ACL
18	Taylor, et al ^22^	10 volunteers	Serum	Sarstedt Monovette serum‐separating tubes	Beckman Coulter CX5 biochemistry analyzer	AL, ALP, ALT, AM, AST, BIC, BUN, Ca^2+^, CK, CK MB isoenzyme, Cl, CREA, DB, GGT, GLU, HDL, K^+^, LDH, LIP, Mg^2+^, Na^+^, Pi, TB, TC, TG, TP, UA	Centrifuged samples within 30 min at RT and Centrifuged samples stored immediately at –80°C	I) Serum samples in contact with the gel stored at 2‐10°C for time periods of 4, 8, 24, 48, 72, 96, 169, 336 h; II) Serum samples stored at 18‐25°C for time periods of 2, 4, 8, 24, 48, 72, 96, 120 h; III) Serum samples stored at 2‐10°C for time periods of 4, 8, 24, 48, 72, 96, 168, 336 h; IV) Serum samples stored at –20°C for periods of 1, 2, 4, 8, 12 weeks; V) Serum samples stored at –20°C with one, two, and three freeze/thaw cycles.	ANOVA or Kruskal‐Wallis test
19	van Balveren, et al ^23^	10 healthy volunteers	Whole blood, plasma	Lithium heparin gel tubes (BD PST II)	Dimension VISTA 1500	AL, ALP, ALT, AM, AST, B12, BIC, BUN, Ca^2+^, Cl, CREA, CRP, DB, FERR, FOL, FT4, GGT, GLU, K^+^, LDH, LIP, Mg^2+^, Na^+^, Pi, TC, TE, TG, troponin I, TSH, UA	Centrifuged samples at 22 ± 1°C	I) Double centrifuged samples after 4 or 8 h from the first centrifugation; II) Uncentrifuged samples until 4 or 8 h after collection stored at 22 ± 1°C.	PD%
20	Zwart, et al ^24^	11 healthy volunteers	Whole blood	Plastic BD Vacutainer® SST™ Plus Blood Collection Tubes (BD)	Not mentioned	AL, ALP, ALT, AST, BSAP, Ca^2+^, Ceruloplasmin, Cl, Copper, COR, CREA, FERR, FOL, Homocysteine, Ir, K^+^, Methylmalonic acid, Na^+^, OST, PTH, Pyridoxal 5′‐phosphate, Retinol‐binding protein, Se, TAC, TC, TF, TF receptors, TG, TP, Transthyretin, vit D, Zinc, α1‐MG, α2‐MG, β‐MG, γ‐MG	Not mentioned	Uncentrifuged samples until 45‐60 min, 2.5, 5, or 24 h after collection stored at RT.	ANOVA, Bonferroni t test

Note

ACL (acceptable change limit) = 1.96 × √2 × CVa.

B (desirable allowable bias) = 0.25 (CVb2 + CVg2) ½.

PD% (percentage deviation) = [(Tx − T0)/T0] × 100 where T0 is the analyte measurement at baseline point, and Tx is the analyte measurement after a given period of time.

RCV (reference change value) = 1.96 × √2 × √(CVa^2^ + CVb^2^).

SCL (significant change limit) = Initial value ± 2.8 usual SD.

TCL (total change limit) = [(2.77 × CVa)2]½ + [(0.5 × CVb)2]½.

CVa, analytical imprecision; CVg, between‐subject variation; CI, confidence interval; ANOVA, one‐way analysis of variance; SEM, standard error of mean; SD, standard deviation; TE, total allowable error; CVb, within‐subject variation.

* The metabolites abbreviations are given in the Analytes Abbreviations section

### 2.4 Data analysis

To quantitatively summarize the reviewed data, we conducted a descriptive statistical analysis. Then, we evaluated the most studied analytes assessed in the least five included papers (Table 2). According to the related articles, we listed the different stability status of each included analyte. Then, we presented the maximum acceptable delays obtained from the relevant articles.

**TABLE 2:** The most thirty‐four studied analytes and related included articles

Analyte	Number of articles related to each analyte	Related articles
ALT	19	^5^,^6^,^7^,^8^,^9^,^11^,^12^,^13^,^14^,^15^,^16^,^17^,^18^,^19^,^20^,^21^,^22^,^23^,^24^
AST	18	^5^,^6^,^7^,^8^,^9^,^10^,^11^,^12^,^13^,^14^,^16^,^17^,^19^,^20^,^21^,^22^,^23^,^24^
K^+^	17	^5^,^8^,^9^,^10^,^11^,^12^,^13^,^14^,^15^,^16^,^17^,^18^,^19^,^21^,^22^,^23^,^24^
TG	17	^5^,^6^,^7^,^9^,^11^,^12^,^13^,^15^,^16^,^17^,^18^,^19^,^20^,^21^,^22^,^23^,^24^
ALP	16	^5^,^7^,^8^,^9^,^11^,^12^,^13^,^15^,^16^,^17^,^19^,^20^,^21^,^22^,^23^,^24^
CREA	16	^5^,^6^,^7^,^9^,^11^,^12^,^13^,^15^,^16^,^17^,^18^,^19^,^21^,^22^,^23^,^24^
TC	16	^5^,^6^,^7^,^9^,^11^,^12^,^13^,^15^,^16^,^17^,^18^,^19^,^21^,^22^,^23^,^24^
AL	15	^5^,^6^,^7^,^11^,^12^,^13^,^15^,^16^,^17^,^18^,^19^,^21^,^22^,^23^,^24^
LDH	15	^5^,^7^,^8^,^9^,^10^,^11^,^14^,^15^,^17^,^18^,^19^,^20^,^21^,^22^,^23^
Na^+^	15	^5^,^8^,^9^,^11^,^12^,^13^,^15^,^16^,^17^,^18^,^19^,^21^,^22^,^23^,^24^
Ca^2+^	14	^5^,^9^,^11^,^12^,^13^,^14^,^15^,^17^,^18^,^19^,^21^,^22^,^23^,^24^
GGT	14	^5^,^6^,^7^,^9^,^11^,^12^,^15^,^17^,^18^,^19^,^20^,^21^,^22^,^23^
TB	13	^5^,^7^,^9^,^11^,^12^,^13^,^15^,^17^,^18^,^19^,^20^,^21^,^22^
BUN	12	^5^,^7^,^9^,^11^,^12^,^13^,^15^,^17^,^19^,^21^,^22^,^23^
CK	12	^5^,^6^,^7^,^9^,^11^,^12^,^15^,^17^,^19^,^20^,^21^,^22^
Pi	12	^5^,^9^,^10^,^11^,^12^,^13^,^15^,^17^,^19^,^21^,^22^,^23^
TP	12	^5^,^6^,^7^,^11^,^12^,^13^,^16^,^17^,^19^,^21^,^22^,^24^
UA	12	^5^,^7^,^9^,^11^,^12^,^13^,^15^,^17^,^19^,^21^,^22^,^23^
AM	10	^11^,^12^,^13^,^15^,^17^,^18^,^19^,^20^,^22^,^23^
Cl	10	^5^,^9^,^12^,^15^,^16^,^17^,^19^,^22^,^23^,^24^
HDL	10	^6^,^7^,^9^,^11^,^12^,^16^,^18^,^19^,^20^,^22^
Mg^2+^	10	^5^,^9^,^10^,^12^,^15^,^17^,^19^,^21^,^22^,^23^
GLU	9	^5^,^7^,^10^,^12^,^14^,^19^,^21^,^22^,^23^
CRP	9	^8^,^9^,^11^,^12^,^15^,^18^,^19^,^21^,^23^
BIC	7	^5^,^9^,^12^,^17^,^19^,^22^,^23^
FERR	7	^9^,^11^,^16^,^18^,^21^,^23^,^24^
Ir	7	^11^,^15^,^16^,^18^,^19^,^21^,^24^
LIP	7	^9^,^11^,^17^,^19^,^21^,^22^,^23^
TF	7	^11^,^15^,^16^,^18^,^19^,^21^,^24^
B12	5	^15^,^18^,^19^,^21^,^23^
COR	5	^15^,^16^,^19^,^21^,^24^
FOL	5	^16^,^18^,^19^,^23^,^24^
FT4	5	^15^,^18^,^19^,^21^,^23^
TSH	5	^15^,^18^,^19^,^21^,^23^

Abbreviations

ALT, Alanine aminotransferase; AST, aspartate aminotransferase; K^+^, potassium; TG, triglyceride; ALP, alkaline phosphatase; CREA, creatinine; TC, total cholesterol; AL, albumin; LDH, lactate dehydrogenase; Na^+^, sodium; Ca^2+^, calcium; GGT, γ‐glutamyltransferase; TB, BUN, total bilirubin; urea; CK, creatine kinase; Pi, inorganic phosphate; TP, total protein; UA, uric acid; AM, amylase; Cl, chloride; HDL, high‐density lipoprotein; Mg^2+^, magnesium; GLU, glucose; CRP, C‐reactive protein; BIC, bicarbonate; FERR, ferritin; Ir, iron; LIP, lipase; TF, transferrin; B12, cobalamin; COR, cortisol; FOL, folate; FT4, free thyroxine; TSH, thyroid‐stimulating hormone.

## 3 RESULTS

**FIGURE 2:**
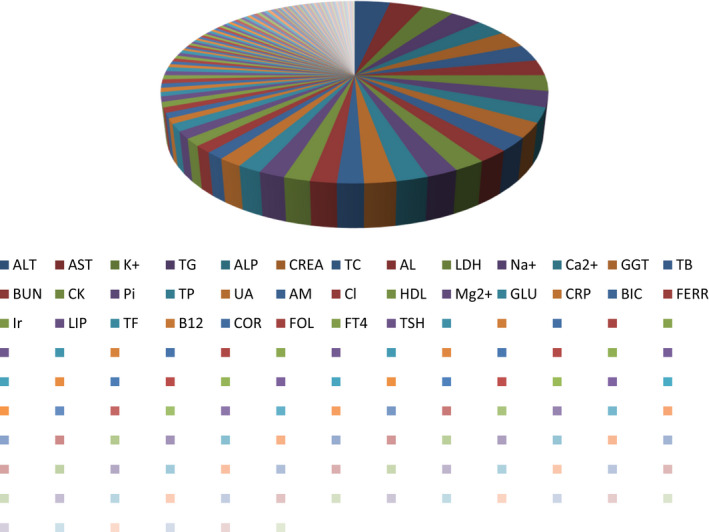
The most thirty‐four studied analytes according to the included papers. Alanine aminotransferase (ALT); aspartate aminotransferase (AST); potassium (K^+^); triglyceride (TG); alkaline phosphatase (ALP); creatinine (CREA); total cholesterol (TC); albumin (AL); lactate dehydrogenase (LDH); sodium (Na^+^); calcium (Ca^2+^); γ‐glutamyltransferase (GGT); total bilirubin (TB); urea (BUN); creatine kinase (CK); inorganic phosphate (Pi); total protein (TP); uric acid (UA); amylase (AM); chloride (Cl); high‐density lipoprotein (HDL); magnesium (Mg^2+^); glucose (GLU); C‐reactive protein (CRP); bicarbonate (BIC); ferritin (FERR); iron (Ir); lipase (LIP); transferrin (TF); cobalamin (B12); cortisol (COR); folate (FOL); free thyroxine (FT4); thyroid‐stimulating hormone (TSH)

In the 20 included references, up to 123 different analytes were assessed. For the accurate evaluations, we focused on analytes investigated in the least 5 papers. Applying the criteria led to the extraction of 34 analytes including ALT, AST, K^+^, TG, ALP, CREA, TC, AL, LDH, Na^+^, Ca^2+^, GGT, TB, BUN, CK, Pi, TP, UA, AM, Cl, HDL, Mg^2+^, GLU, CRP, BIC, FERR, Ir, LIP, TF, B12, COR, FOL, FT4, and TSH (Table 2 and Figure 2).

Most studies used healthy subjects instead of patient samples. The sample size was ranged between 6 and 156 individuals. Temperature studied for analyte stability defined as 4 statues: (1) room temperature (15‐35°C), (2) refrigerated temperature (4‐8°C), (3) deep freezer (−20°C), and (4) deep freezer (−80°C). According to the included studies, the lower studied storage time was 45 min and the higher studied storage time was 12 month. Long‐term storage of analytes has been investigated in biobank studies. Maximum allowable difference specifications were based on biological and/or statistical variation.

For the 34 biochemistry analytes, the maximum acceptable delays obtained from the relevant articles (Table 2) are as follows. The analytes are presented in order of the analyte frequency evaluations in studies. It should be noted that the presented maximum acceptable delays might not be universally reproducible because they are according to the defined approaches of the included studies^25^ (Table 1). Most analytes were stable at −20 or −80°C such that some studies had used the −80°C storage condition as a baseline value.^11^, ^20^, ^22^ Hereupon, we focused on a basic classification set as refrigerated or room temperature (RT).

### 3.1 Alanine aminotransferase (ALT)

Nineteen of the 20 articles studied the ALT stability in different storage conditions. Accordingly, ALT activity changed substantially at room temperature. However, it could be stable up to 7 days in chilled blood.

### 3.2 Aspartate aminotransferase (AST)

AST, the second most frequently studied analyte, was stable at least up to 56 hours in whole blood samples and up to 2 weeks in fridge serum.

### 3.3 Potassium (K^+^)

The maximum stability for K^+^ was 24 hours in whole blood at RT. The increase in K^+^ after 24 hours was attributable to Na^+^/K^+^‐ATPase pump failure, with diffusion of K^+^ from the erythrocytes.

### 3.4 Triglyceride (TG)

Seventeen of the 20 articles studied the TG stability in different storage conditions. Accordingly, it could be measured reliably in whole blood samples kept at room temperature for at least a week before plasma separation. TG in fridge serum samples was stable for two weeks.

### 3.5 Alkaline phosphatase (ALP)

ALP activity was stable either in serum separator gel tube or in plain tube and could be measured reliably in serum samples kept at RT or at 4°C for at least 3 days.

### 3.6 Creatinine (CREA)

One‐week delay for chilled whole blood samples and two‐week delay for fridge serum samples were the reported maximum acceptable delays for CREA.

### 3.7 Total cholesterol (TC)

Total cholesterol, such as TG, is a stable analyte and could be measured reliably in whole blood samples kept at room temperature for at least a week. TC in fridge serum samples was stable for two weeks.

### 3.8 Albumin (AL)

One‐week delay for whole blood samples at RT and two‐week delay for fridge serum samples were the maximum acceptable delays for AL that has been reported by the included studies.

### 3.9 Lactate dehydrogenase (LDH)

LDH activity was stable as long as one week in the serum gel tube at both RT and 4°C.

### 3.10 Sodium (Na^+^)

Fifty‐six‐h delay for whole blood samples at RT and two‐week delay for fridge serum samples were the maximum acceptable delays for Na^+^ that have been reported by the included studies.

### 3.11 Calcium (Ca^2+^)

The maximum acceptable delay for Ca^2+^ was 24 hours storage at 4°C and RT in whole blood and in plasma or serum samples.

### 3.12 γ‐glutamyltransferase (GGT)

For GGT, there are 14 reports of either slight or prominent changes in its activity. The maximum stability reported for GGT was 7 days at 4°C.

### 3.13 Total bilirubin (TB)

The percentage changes in total bilirubin were not clinically meaningful when stored in serum gel tubes either at RT or chilled up to one week.

### 3.14 Urea (BUN)

Urea in plasma and serum that were exposed to prolonged contact with cells and in double‐spun specimens remained stable over the 56‐h period. It was stable up to 30 days at −20°C.

### 3.15 Creatine kinase (CK)

For CK, there are 12 reports of either slight or prominent changes in its activity. The maximum stability reported for CK was 7 days at 4°C.

### 3.16 Inorganic phosphate (Pi)

A number of studies have reported a significant decrease in phosphate concentration in whole blood samples in the early hours after phlebotomy due to glycolysis.^1^, ^13^ However, most studies have reported an increase in Pi concentration due to hydrolysis of intraerythrocytic phosphate esters and leakage of the inorganic part.^1^, ^15^ Therefore, Pi is one of the most unstable analytes.

### 3.17 Total protein (TP)

Concentrations of total protein did not differ either in serum separator gel tube or in plain tube and could be measured reliably in samples kept at RT or at 4°C for at least three days after centrifugation. It was also stable in whole blood samples up to one week at RT.

### 3.18 Uric acid (UA)

For uric acid, gel tubes showed enhanced stability compared to plain tubes and the stability can be maximized by refrigeration at 4°C.

### 3.19 Amylase (AM)

The results of amylase were somewhat contradictory. Most studies have found that AM is generally very stable when it was stored at 4°C or below. However, one study showed decreased levels of serum AM in prolonged storage at −20°C.

### 3.20 Chloride (Cl)

Chloride could be measured reliably in whole blood and in plasma/serum samples kept at room temperature for at least 24 hours.

### 3.21 High‐density lipoprotein (HDL)

HDL was very stable when stored below 4°C, but some fluctuations were noted at 25°C. Nevertheless, it has been demonstrated that HDL could be measured reliably in whole blood samples kept at room temperature for at least a week before plasma separation.

### 3.22 Magnesium (Mg^2+^)

Magnesium stability in whole blood was dependent on storage temperature. It is less stable at RT (a few hours) and more stable at 4°C (up to 48 hours). In double‐spun plasma and serum centrifuged immediately, it was reported that Mg^2+^ could be stable up to 56 hours.

### 3.23 Glucose (GLU)

Glucose was an unstable analyte that could only be kept stable at RT and 4°C up to 24 hours using tubes containing glycolysis inhibitors.

### 3.24 C‐reactive protein (CRP)

CRP was unaffected when stored at 4°C for up to 72 hours after centrifugation. It was stable up to 24 hours when stored at RT in whole blood samples.

### 3.25 Bicarbonate (BIC)

Bicarbonate was one of the analytes that was not stable after centrifugation and its amount decreased in plasma samples.

### 3.26 Ferritin (FERR)

Ferritin could be measured reliably in whole blood samples kept at RT for at least 24 hours before cell separation. But it did not remain stable at higher temperatures.

### 3.27 Iron (Ir)

Iron was stable after 24 hours storage at 4°C and RT in whole blood and in serum/plasma samples.

### 3.28 Lipase (LIP)

Except for decreased activity at temperature fluctuation events, lipase was stable in all analytical circumstances studied.

### 3.29 Transferrin (TF)

Transferrin was stable in all analytical circumstances studied.

### 3.30 Cobalamin (B12)

Cobalamin was not significantly affected up to 72 hours in whole blood and serum or plasma at RT and refrigerated temperatures in plain glass and any kind of tubes.

### 3.31 Cortisol (COR)

Cortisol also did not significantly change up to 72 hours in whole blood and serum or plasma at RT and refrigerated temperatures in plain glass and any kind of tubes.

### 3.32 Folate (FOL)

In decapped plasma samples stored at RT, folate was stable for 2 hours. In whole blood, folate was not significantly affected up to 72 hours at 4°C and up to 48 hours at 25°C. It is stable in serum or plasma at room or refrigerated temperatures up to 72 hours.

### 3.33 Free thyroxine (FT4) and Thyroid‐stimulating hormone (TSH)

The same five articles studied FT4 and TSH stabilities. FT4 and TSH hormones were not significantly affected up to 72 hours in whole blood and serum or plasma at room or refrigerated temperatures. The two hormones were also stable at 35°C until 24 hours. In decapped plasma samples stored at RT, FT4 was stable for 4 hours and TSH was stable for 6–8 hours.

## 4 DISCUSSION

A comprehensive discussion about studies reached to the different results is complicated. Different panels of analytes, measured in different matrices, collected from different populations, variety in sample sizes, different storage times, different temperature conditions, different technologies and analyzers, different acceptance limits, or statistical models are the papers variables that make it difficult to obtain an intact output and conclusion. However, dividing the problem into its components would probably lead to solve the puzzle.

### 4.1 Type of samples

Key differences between plasma and serum such as the presence of fibrinogen and the other clotting factors, or the need to use anticoagulant agents such as ethylenediaminetetraacetic acid (EDTA) or heparin, probably affects the analytes’ stability. In Boyanton et al study BIC, TP, Ca^2+^, ALT, TC, Mg^2+^, and GGT showed significant changes in plasma samples, while they were stable in serum up to 56 hours.^5^

The choice of sample type for doing biochemical evaluations depends on the analyte to be determined. For example, toxic trace elements as lead, cadmium, and mercury are bonded preferably on erythrocytes, and therefore, for its determination, the whole blood is preferred. However, for Pi, ALT, AST, ALP, and many others biochemical analytes serum is preferred (https://www.labcorp.com/test‐menu/search). On the other hand, samples collected in tubes with EDTA were practical for the measurement of hormones because EDTA is known to protect peptides from proteolysis.^19^, ^26^

### 4.2 Type of blood collection tube

Blood collection tubes consist of tube walls, rubber stoppers, lubricants, anticoagulants, separator gels, clot activators, and surfactant, all of which can affect the quality of the specimens, accuracy, and precision of laboratory tests.^27^

Some analytes were significantly affected by tube type such as K^+^, Pi, and Mg^2+^.^10^, ^19^ Gawria et al have been reported that potassium and phosphate were clearly more stable in the mechanical separator tubes (Barricor™) than in the gel tubes.^10^ Cuhadar et al also found that some restrictions must be applied for GLU, AST, BUN, HDL, and UA analyses in serum gel or non‐gel.^7^ Glucose concentrations decreased with increasing serum clot contact time in the plain tube, because of glycolytic action of erythrocytes and leukocytes.^7^ However, gel tubes especially tubes containing sodium fluoride showed enhanced stability compared to plain tubes and the stability could be maximized up to 24 hours by refrigeration.^10^, ^14^, ^19^

### 4.3 Delays before centrifugation

Plasma and serum should ideally be separated from cells as quickly as possible to prevent cellular metabolism and analytes movement between cellular parts and the plasma or serum. Prolonged contact of plasma or serum with blood cells occurring by delays before centrifugation is a common cause of false test results.^5^ Before the centrifugation, the storage time and temperature the whole blood samples were stored in are very important items for reduction of the false results. According to the reviewed papers, K^+^, Pi, Mg^2+^, Ca^2+^, Ir, LDH, GLU, CREA, BUN, and FERR are the analytes most influenced by delayed centrifugation, because they are present in cells and they leak out of the cell over the time.^19^, ^21^

Temperature and time storage of whole blood samples are important for improving the analyte stability. Although some analytes such as Pi, Mg^2+^, and GLU are not stable in whole blood at RT for more than 24 hours, they become more stable when samples stored at 4°C compared to RT.^19^, ^28^

### 4.4 Delays after centrifugation

At clinical laboratories especially hospital laboratories, serum/plasma samples are often stored for a given time for potential later analysis. Knowing about the reliability and stability of analytes in samples stored at RT or lower temperatures for reanalysis is very important.

According to the reviewed studies, LDH and bicarbonate were the analytes with the lowest stability after centrifugation; therefore, any reanalysis of these analytes in centrifuged tubes cannot be allowed.^9^ However, many analytes in the double‐spun specimens could be stable for up to 56 hours at ambient temperature.^5^

## 5 CONCLUSION

Given the results of this study, analytes including K^+^, Pi, Mg^2+^, GLU, AST, BUN, HDL, and UA have different stability depending on the tube type selected. K^+^, Pi, Mg^2+^, Ca^2+^, Ir, LDH, GLU, CREA, BUN, and FERR have variable stability when centrifugation is delayed. Any reanalysis of analytes including LDH and bicarbonate in centrifuged tubes are not recommended.

It is necessary to emphasize that this study only combines the results of 20 stability studies and summarizes their outcomes systematically to generate maximum analyte stability information. This study may be useful for definition of acceptable delay times and temperatures when pre‐analytical sample handling takes longer than usual. However, there are some discrepancies between the results of the included studies may reflect differences in the analytical methodology. A meta‐analysis study is recommended for future studies to estimate the effect of storage conditions on biochemical testing.
